# Serum Endocan as a Predictor of Survival and Cardiovascular Events in Patients Without Diabetic Kidney Disease on Chronic Haemodialysis: A Prospective, Observational Study

**DOI:** 10.3390/medicina61060991

**Published:** 2025-05-27

**Authors:** Mario Šafer, Ivan Feldi, Ines Šahinović, Ivana Tolj, Marko Pirić, Dunja Šojat, Eduard Oštarijaš, Dubravka Mihaljević

**Affiliations:** 1 Department of Internal Medicine, General Hospital Virovitica, Lj. Gaja 21, 33000 Virovitica, Croatia; mario.safer27@gmail.com; 2 Faculty of Medicine Osijek, Josip Juraj Strossmayer University of Osijek, J. Huttlera 4, 31000 Osijek, Croatiaivanatolj5@gmail.com (I.T.);; 3 Department of Internal Medicine, General County Hospital Našice, 31500 Našice, Croatia; 4 Clinical Institute for Laboratory Diagnostics, Clinical Hospital Centre Osijek, J. Huttlera 4, 31000 Osijek, Croatia; 5 Department of Nephrology, Clinic of Internal Medicine, Clinical Hospital Centre Osijek, J. Huttlera 4, 31000 Osijek, Croatia; 6 Health Centre of Osijek-Baranja County, Park Kralja Petra Krešimira IV. 6, 31000 Osijek, Croatia; 7 Doctoral School of Clinical Medical Sciences, Medical School, University of Pécs, Szigeti út 12, 7624 Pécs, Hungary

**Keywords:** endothelial dysfunction, haemodialysis, endocan, MACE

## Abstract

*Background and Objectives*: Chronic kidney disease (CKD) is an increasingly significant global public health issue, with cardiovascular disease being the leading cause of mortality. Endothelial dysfunction plays a critical role, but diagnostic tools have certain limitations. Endocan, a soluble proteoglycan, emerged as a promising endothelial dysfunction marker and potential major adverse cardiovascular event (MACE) predictor in haemodialysis (HD) patients. *Materials and Methods*: In this single-centre, observational, prospective study, non-diabetic HD patients without prior MACEs were monitored. A total of 75 participants met the inclusion criteria. We measured serum endocan, standard biochemical and anthropometric parameters, and parameters of peripheral and central haemodynamics before and after HD in all participants. *Results*: Patients with higher endocan were older, had elevated CRP and reduced albumin concentrations, and often had a tunnelled central venous catheter (TCVC) for vascular access. Higher serum endocan levels were independently associated with an increased risk of MACEs (aHR = 4.09, 95%-CI: 1.72–9.74), MACE-related mortality (aHR = 2.64, 95%-CI: 1.23–5.66), and all-cause mortality (aHR = 1.86, 95%-CI: 1.07–3.23), both before and after adjusting for predefined confounders, with the highest endocan tercile exhibiting the shortest event-free survival. *Conclusions*: Endocan is a valuable marker of inflammation and endothelial dysfunction in non-diabetic HD patients. Its elevated concentration indicates an increased cardiovascular risk and more frequent MACEs. Future multicentre studies with repeated endocan assessments should validate its prognostic and diagnostic utility, particularly in long-term patient follow-up.

## 1. Introduction

Chronic kidney disease (CKD) is a growing global public health concern, with a prevalence of approximately 8% to 16% [1]. Cardiovascular disease (CVD) is the primary cause of morbidity and mortality in both CKD and end-stage kidney disease (ESKD) [2,3]. Approximately, 2 out of 1000 patients with CKD (0.2%) have ESKD and are on dialysis [4]. Mortality among individuals on dialysis is high, and sudden cardiac arrest (SCA) accounts for a large percentage, with estimates ranging from 14% to 45% [5,6,7]. In patients undergoing dialysis, SCA events were significantly more common on dialysis days and 3-fold higher than expected by chance [8]. Diabetic kidney disease is the leading cause of ESKD, accounting for 30% to 50% of the incident cases in the United States and most developed countries [9], but diabetes itself is also an independent risk factor for CKD progression and CVD. Among various traditional and non-traditional risk factors linking CKD and CVD, endothelial dysfunction stands out as a key contributor, which manifests as endothelial cell activation leading to vasoconstriction, inflammation, and a pro-atherothrombotic state [10,11]. Current methods of assessing endothelial dysfunction—ranging from invasive coronary flow measurements to non-invasive flow-mediated dilation (FMD)—have limitations due to invasiveness, high costs, and technical demand [12]. Hence, there is a need for novel biomarkers of endothelial dysfunction.

Endocan, a proteoglycan secreted by activated endothelial cells, has emerged as a promising marker of endothelial dysfunction. It is involved in regulating cell adhesion, migration, proliferation, and neovascularisation, and it has shown promising utility in various kidney disease entities, including ESKD [13,14,15,16,17,18,19]. Higher endocan levels have been associated with major endothelial damage, the progression of CKD, an increased likelihood of transplant rejection, and higher rates of cardiovascular events (CVEs) and mortality [20,21]. Although previous studies have investigated the role of endocan in this context, its role specifically in patients without diabetes but with CKD remains less explored.

In this study, we specifically measured serum levels of endocan and parameters of central and peripheral haemodynamics in non-diabetic HD patients without prior major adverse cardiovascular events (MACEs) to investigate its potential as a marker of vascular dysfunction independent of diabetes. Additionally, the study examined how endocan is linked to MACEs and multiple survival parameters over the follow-up period. A MACE is a composite clinical endpoint including myocardial infarction, stroke, hospitalisation for heart failure, and cardiovascular death.

## 2. Materials and Methods

### 2.1. Study Population and Design

This is a single-centre, non-randomised, observational, prospective cohort study that was conducted from March 2018 until March 2023 at the Department of Nephrology, Clinical Hospital Centre Osijek, Croatia. A total of 75 patients of both genders who met the inclusion criteria were included in the research. The inclusion criteria were as follows: the participants were older than 18 years, had been on haemodialysis for at least 3 months, were dialysed 3 times a week for 4 h using the haemodiafiltration modality, and had to be anuric with a maximum urine output of up to 200 mL in 24 h. Also, their Kt/V, as a measure of dialysis adequacy, had to be >1.4. Patients were excluded if they were undergoing active oncologic treatment or if less than five years had passed since the completion of cancer therapy; if they had atrial fibrillation with rapid ventricular response accompanied by symptomatic heart failure at the time of enrolment; if they had undergone lower limb amputation; if they had active symptoms or a confirmed and treated systemic inflammatory or infectious disease at the time of enrolment; if they had diabetes mellitus; or if they had a history of MACEs. A MACE was defined as acute coronary syndrome, cerebrovascular insult, or hospitalisation due to an episode of acute heart failure. Each patient was informed about all protocols and procedures of this research, and all participants signed an informed consent form for participation, which adhered to all modern ethical and deontological principles. The study protocol adhered to the standards established by the latest revision of the Declaration of Helsinki and was approved by the Ethics Committee of the Faculty of Medicine Osijek and the Ethics Committee of the University Hospital Osijek (Number: R2-6782/2018, date of approval 3 May 2018). The study was conducted at the Department of Nephrology and the Clinical Institute for Laboratory Diagnostics, University Hospital Osijek.

### 2.2. Demographic Data, Anthropometric Analyses, and Biochemical Investigations

We analysed routine biochemical parameters, such as complete blood count, C-reactive protein (CRP), total cholesterol, low-density lipoprotein (LDL) cholesterol, glucose, albumin, calcium, phosphorus, parathyroid hormone (PTH), ferritin, and iron. These parameters were measured using standard methods. Plasma samples were collected using tubes treated with ethylenediaminetetraacetic acid (EDTA) on the day of enrolment in the study. Following centrifugation for 15 min at 1000× *g* at room temperature, the samples were stored at −80 °C until analysis. A separate blood sample for serum analysis of endocan was obtained using commercially available enzyme-linked immunosorbent assay (ELISA) kits (using Magnetic Luminex^®^ Screening Assay multiplex kits, R&D Systems, Minneapolis, MN, USA) at the Clinical Institute for Laboratory Diagnostics, Clinical Hospital Centre Osijek, Croatia.

Blood samples for biochemical analysis were obtained just before a midweek HD session (Wednesday or Thursday) together with measurements of parameters of central and peripheral haemodynamics. Hydration status is most stable before midweek sessions (e.g., Wednesday or Thursday), reducing variability due to fluid overload or excessive dehydration. Blood pressure and vascular tone are more consistent compared to post-dialysis, when vasodilation and volume shifts occur. Also, reproducibility is highest in this pre-dialysis, midweek window, making it the preferred standard.

Each participant was weighed before HD, and the total interdialytic weight gain (IWG) was recorded in relation to the estimated dry weight, which was assessed using the bioimpedance method with a body composition monitor (BCM) device (BCM, Fresenius Medical Care, Bad Homburg, Germany). Demographic data and medical history were obtained from the medical records of each participant. Accordingly, their age, gender, HD vintage, vascular access, aetiology of CKD, history of MACEs, malignant disease, inflammatory diseases, and diabetes were recorded.

MACEs, cardiovascular mortality, and all-cause mortality were the investigated outcomes. The cause of mortality was assessed from medical records and through interviews with attending physicians and family members. Participants who underwent kidney transplantation during the follow-up period were excluded from the study.

### 2.3. Assessments of Peripheral and Central Haemodynamic Parameters

Two consecutive measurements of pulse wave velocity (PWV), peripheral systolic and diastolic blood pressure (SBP and DBP), central aortic SBP and DBP, heart rate (HR), mean arterial pressure (MAP), pulse pressure (PP), and augmentation index (AI) were performed using a Mobil-O-Graph Agedio B900 device (IEM GmbH, Aachen, Germany) before and after a midweek HD session. The rationale for this was that immediately after dialysis, blood pressure drops, vessels dilate, and arterial stiffness can be underestimated, whereas the day after a long dialysis break, patients are often fluid overloaded, which can falsely increase arterial stiffness.

This non-invasive oscillometric device utilises integrated ARCSolver algorithms (Austrian Institute of Technology, Vienna, Austria) to derive aortic stiffness surrogates based on brachial blood pressure readings through a generalised transfer function. Measurements were performed in the supine position after 5 min of rest, using cuffs adapted to the circumference of the non-fistula arm. Blood samples for biochemical analysis were then obtained, and patients began their HD session. After 4 h of HD and a 5 min rest in the supine position, measurements were repeated following the same protocol.

### 2.4. Statistical Analysis

We summarised demographic, biochemical, and clinical characteristics across serum endocan levels stratified by terciles using descriptive statistics, with categorical variables compared using the chi-square test with continuity correction, normally distributed continuous variables using one-way ANOVA, and non-normally distributed continuous variables using the Kruskal–Wallis test. Count data were presented as a whole number (percentage), and normally distributed variables were presented as the mean ± standard deviation (SD), whereas non-normally distributed variables were expressed as the median [Q1, Q3]. The association of serum endocan levels with MACEs and mortality was assessed by a time-to-event analysis using Kaplan–Meier survival curves and Cox proportional hazards multivariate models, with serum endocan levels analysed both as a continuous variable and stratified into terciles. Hazard ratios (HRs) with 95% confidence intervals (95%-CI) were reported from the multivariable Cox models, adjusted for the predefined potential confounders selected based on clinical expertise: age, sex, HD vintage, albumin, BMI, central SBP, and AI. All analyses were performed in R version 4.4.1 (R Core Team, Vienna, Austria).

## 3. Results

### 3.1. Demographic, Clinical, and Biochemical Characteristics

**Figure 1:**
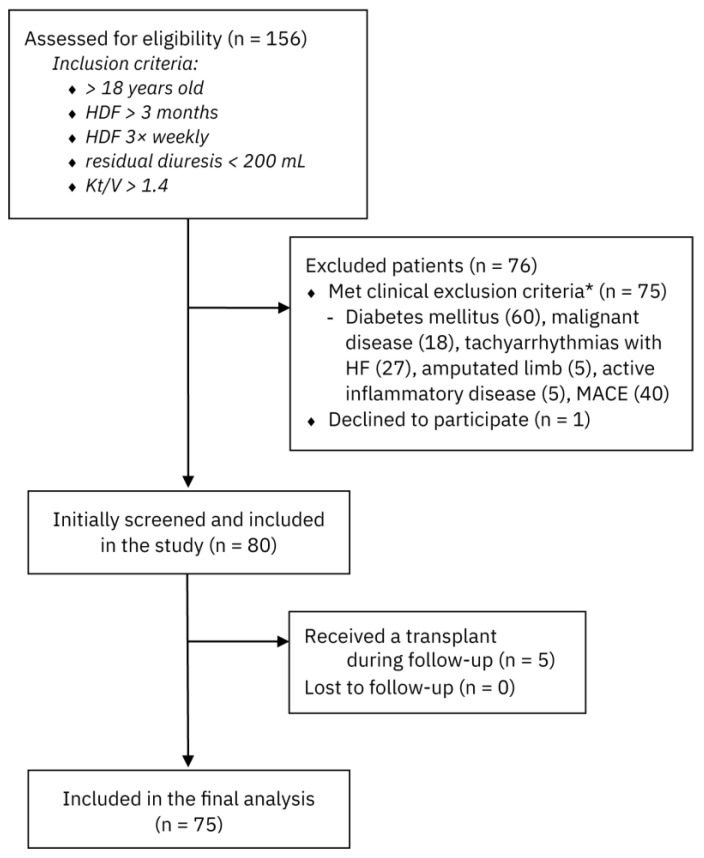
Participant selection process. HDF: haemodiafiltration; MACE: major adverse cardiovascular event. Note: * The sum of individual exclusion criteria exceeds the total number of excluded patients, as some patients fulfilled multiple exclusion criteria simultaneously.

The patients were enrolled in the study between March 2018 and March 2019 and were followed until March 2023. The median follow-up period was 40 [18.5, 60] months. During this time, a total of 156 patients were initially screened for eligibility. Following the exclusion of individuals who either did not meet the inclusion criteria or declined participation, 80 patients were ultimately enrolled for analysis and follow-up. A total of five patients received a kidney transplant during follow-up and were, therefore, excluded from further analyses. A flow diagram outlining the participant selection process is presented in Figure 1.

A total of 75 participants were included in the study and stratified into terciles based on serum endocan concentrations. Of these, 31 (41.3%) were women. The overall median age was 70 [59, 76.5] years, the median dialysis vintage was 52 months [21.5, 108.5], and there were 34 patients (45.33%) with a tunnelled central venous catheter (TCVC) for vascular access. Other, detailed baseline demographic, biochemical, and clinical characteristics of the study population, stratified by serum endocan concentrations, are reported in Table 1. Patients with higher levels of endocan were older (50.00 [41.00, 64.00], 71.00 [64.00, 75.00], and 77.00 [72.00, 81.00] years for first, second, and third terciles, respectively) and more frequently had a TCVC. The TCVC was significantly lower only in the first tercile, where there was a significantly higher number of patients with arteriovenous fistulas (AVFs). Other indicators such as gender, HD vintage, IWG, dialysis dose, aetiology of CKD, and the use of antihypertensive and hypolipemic medications were not statistically significantly associated with endocan levels.

Regarding biochemical parameters, patients with higher endocan levels had significantly higher levels of CRP and lower serum albumin concentrations. Statistical significance for CRP was found between the first and third terciles, as well as between the second and third terciles. Statistical significance for albumin was noted only between the first and second terciles. The numbers of leukocytes, erythrocytes, platelets, haemoglobin, iron, ferritin, calcium, phosphorus, PTH, total cholesterol, and LDL cholesterol were not statistically associated with different serum endocan concentrations. A detailed analysis of all investigated variables can be found in Table 1, along with the notation of statistical significance.

### 3.2. Parameters of Peripheral and Central Haemodynamics

Peripheral and central haemodynamic parameters of the study population, according to serum endocan concentration, are summarised in Table 2. In the measurements taken before HD, higher values according to terciles were observed for peripheral SBP, MAP, PP, central aortic SBP, and central PP, with statistically significant differences found between the first and third terciles. For the AI, statistical significance was noted between the second and third terciles, as well as between the first and third terciles, while PWV was statistically significantly elevated across all terciles. In measurements taken after HD, both PWV and the AI retained statistical significance between all terciles. There were no statistically significant differences among the other parameters. A detailed analysis of all investigated variables can be found in Table 2, along with the notation of statistical significance.

### 3.3. Outcomes and Survival

The total average follow-up period was 40 months [18.5, 60]. During the follow-up period, a total of 32 MACEs were recorded, of which 20 out of 32 (62.5%) occurred in the third tercile of serum endocan levels, which was statistically significant. Of the total number of patients who started the study, 46 out of 75 (61.33%) died; 29 out of 46 (63.04%) died from MACEs, while 17 out of 46 (36.96%) died from other causes. Among the survivors, 21 out of 29 (72.41%) were in the first tercile of serum endocan levels, while 20 out of 29 (68.97%) of those who died from MACEs were in the third tercile, which was statistically significant. A detailed analysis of all investigated variables can be found in Table 3, along with the notation of statistical significance.

**Figure 2:**
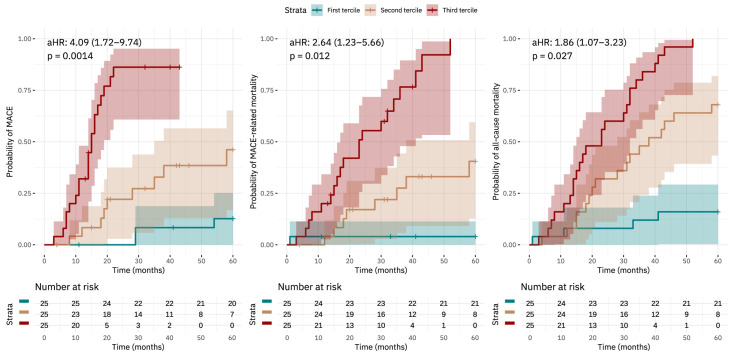
Kaplan–Meier survival curves showing probability of MACEs, MACE-related mortality, and probability of all-cause mortality, shown alongside with the Cox proportional hazards multivariate model results.

Time-to-event analysis using Kaplan–Meier survival curves and Cox proportional hazards multivariate models adjusted for the predefined potential confounders (i.e., age, sex, HD vintage, albumin, BMI, central SBP, and AI) are reported in Figure 2. One initially preselected variable (pulse wave velocity) was excluded from the final multivariate model due to a very strong collinearity with serum endocan levels (Spearman’s rho = 0.82, *p* < 0.0001) in order to not compromise model stability and interpretability. Higher serum endocan levels were independently associated with an increased incidence of MACEs (aHR = 4.09, 95%-CI: 1.72–9.74, *p* = 0.0014), MACE-related mortality (aHR = 2.64, 95%-CI: 1.23–5.66, *p* = 0.012), and increased all-cause mortality (aHR = 1.86, 95%-CI: 1.07–3.23, *p* = 0.027), both before and after adjusting for predefined confounders, with the highest endocan tercile exhibiting the shortest event-free survival. Detailed model results with all variables are displayed in Table 4. To assess the robustness of the association, we additionally utilised a reduced model constructed via stepwise variable selection based on univariate significance (*p* < 0.05), in which endocan levels remained statistically significant.

## 4. Discussion

Our main findings are that patients with higher serum endocan levels had an increased risk of MACEs, MACE-related mortality, and all-cause mortality, both before and after adjusting for predefined confounders, with the highest endocan tercile exhibiting the shortest event-free survival in the population of patients without DKD and prior MACEs.

Our study is distinguished from previous research by the exclusion of patients with DKD and those with a history of MACEs prior to enrolment; such an approach ensured a unique study population. Given that earlier studies have demonstrated the substantial impact of diabetes and prior MACEs on endocan concentrations and future cardiovascular outcomes, our results further reinforce the significance of endocan as a potential biomarker that can be used as an independent cardiovascular risk factor in HD patients.

Other studies have also investigated the impact of endocan on the occurrence of MACEs and survival in populations of patients with CKD, those on HD, and those on PD. Several studies in CKD and PD populations demonstrated that serum endocan is associated with survival and both fatal and non-fatal MACEs [17,22]. In a population of patients on HD, Kim et al. evaluated the potential predictive role of endocan for cardiovascular risk in HD patients. The high-endocan group experienced more frequent MACEs, and endocan served as an independent predictor of MACEs in patients on HD [23]. Lin et al. established a correlation between higher endocan levels and all-cause mortality in HD patients [24]. Wu et al. concluded that among patients on maintenance HD, serum endocan levels positively correlated with the surrogate marker of aortic stiffness, both in a dose-dependent manner and with significant predictive power [25]. It is important to reiterate that the studies conducted by Kim et al., Wu et al., and Lin et al. included participants with diabetes and those with a prior history of MACEs, both of which are independent risk factors for (subsequent) MACEs.

In CKD, traditional risk factors cannot explain the high prevalence of CVD disease [26]. Factors related to endothelial dysfunction have increasingly been studied [21]. Several studies have demonstrated that endocan is involved in endothelial dysfunction and inflammation and could be an independent risk factor for poor clinical outcomes in CVD and CKD [23,27,28,29].

We showed that patients with higher serum concentrations of endocan were older, had higher levels of CRP, and lower albumin levels. It is expected that arteries will be stiffer and that endothelial dysfunction will be more pronounced with aging. Persistent inflammation results in a lower albumin concentration and leads to the deterioration of endothelial dysfunction. Statistically significant declines in albumin levels begin in the second tercile of endocan concentration, suggesting that patients enter a catabolic phase and a state of chronic inflammation relatively quickly. Similar findings were reported by other authors, albeit in populations of CKD patients who were not yet on HD and in patients on PD. For instance, Yilmaz et al. studied plasma endocan levels in non-dialysed CKD patients at stages 1–5 (n = 215) in relation to inflammation, endothelial dysfunction, MACE incidence, and overall survival. They found that plasma endocan concentration correlated with the estimated glomerular filtration rate, different markers of inflammation (pentraxin 3 and high-sensitivity CRP (hs-CRP), FMV, and carotid intima–media thickness (CIMT) [22]. Pawlak et al. conducted a cross-sectional study in non-dialysed CKD patients (n = 53) with and without CVD, aiming to evaluate plasma endocan, soluble intercellular adhesion molecule-1 (sICAM-1), soluble vascular adhesion molecule-1 (sVCAM-1), markers of inflammation (hs-CRP, interleukin-6, and tumour necrosis factor-α), and their inter-relations. They found that plasma endocan, sICAM-1, sVCAM-1, and hs-CRP levels were significantly higher in CKD patients than in controls and also significantly higher in CKD patients with CVD than in those without CVD [29]. Poon et al. examined the relationship between serum endocan level and clinical outcomes of PD patients (n = 193). The results revealed that patients with higher serum endocan levels in tercile 3 had lower serum albumin levels, higher carotid–femoral PWV, and higher CRP than those in terciles 2 and 1. Similar to our findings, they observed a close correlation between serum endocan and CRP levels, suggesting that endocan may be involved in systemic inflammation, a notion supported by the recent literature indicating that it partly modulates leukocyte diapedesis [17,22,23].

In the group of patients with higher endocan, we observed that TCVCs were more frequently used for vascular access to HD. Statistically, this was significant for the first tercile, where AVFs dominated. We hypothesise that patients with an AVF had lower PWV values and, therefore, lower arterial stiffness, which would, therefore, lower the serum endocan levels. In the existing literature, we did not find data on vascular access in relation to endocan levels and endothelial dysfunction.

The unexpectedly high prevalence of central venous catheters in this cohort likely reflects centre- and population-specific factors, including late referral to nephrology care: patients who begin dialysis without prior nephrology follow-up often start with a catheter due to the absence of a mature AVF or a graft. We also hypothesise that older patient populations with limited life expectancy or poor vascular access options may be maintained on catheters instead of undergoing AVF creation. In our study, it is evident that patients in the second and third tertiles (where CVCs are most prevalent), were statistically significantly older.

Among the parameters of peripheral and central haemodynamics, an important factor in progression of CVD and CKD is arterial stiffness. PWV has become a reliable indicator for evaluating arterial stiffness and serves as an independent predictor of cardiovascular and all-cause mortality in various populations, including those with ESKD [30,31,32]. We demonstrated that PWV increases with terciles of ndocan concentration, which aligns with the inter-related pathophysiological connection where endothelial dysfunction contributes to the development of arterial stiffness and vice versa. Similar findings have been reported in populations of patients with CKD, as well as in those on PD and HD [17,22,23]. Since endocan has been reported to affect CIMT and FMV [22,33], it is not surprising to observe a similar relationship with arterial stiffness.

In patients on HD without diabetes mellitus, the pathophysiological mechanisms driving CVEs may be less influenced by metabolic factors and more dependent on endothelial dysfunction, inflammation, and arterial stiffness, namely the pathways in which endocan is believed to play a role. Endocan reflects endothelial cell activation, a central feature in atherosclerosis, arterial stiffness, and vascular calcification, all of which are common in CKD and HD populations [23]. However, in non-diabetic patients, where glycaemic endothelial damage is absent, elevated endocan may more directly reflect non-metabolic endothelial injury (e.g., from uraemia, oxidative stress, fluid overload, or other mechanisms). Endocan modulates leukocyte adhesion and transmigration, promoting vascular inflammation, which is a known trigger for plaque instability and CVEs. This effect is independent of diabetic status and may be more pronounced in HD patients with a high inflammatory burden [22]. Studies suggest a link between elevated endocan levels and increased PWV, a surrogate marker of arterial stiffness and a strong independent predictor of cardiovascular mortality in HD patients [25]. In non-diabetic HD patients, elevated PWV and endocan may synergistically identify high-risk vascular phenotypes, even in the absence of traditional risk factors like hyperglycaemia.

The clinical application of endocan extends across several domains. As a marker of endothelial activation and vascular inflammation, endocan may serve in the early detection of subclinical vascular damage in high-risk groups such as patients with CKD, ESKD, diabetes, and hypertension. In the context of maintenance HD, where traditional risk markers often lack sensitivity, endocan may aid in risk stratification and longitudinal monitoring, providing insight into ongoing vascular injury. Additionally, given its correlation with arterial stiffness, endocan could be used as a complementary tool alongside PWV in the assessment of vascular aging. Also, its sensitivity to pro-inflammatory stimuli supports its potential role in monitoring systemic inflammation in CKD, ESKD, or autoimmune disease populations, where low-grade chronic inflammation significantly contributes to disease progression and adverse cardiovascular outcomes. Finally, it is important to mention the review article by Samouilidou et al. and the meta-analysis by Khalaji et al. which suggest that recent studies reveal that endocan could be a predictor of all-cause mortality and MACEs in CKD patients. Endocan levels were found to be higher in patients with CKD compared to healthy controls, despite high heterogeneity, and were also demonstrated as a negative prognostic factor in patients on HD, PD, and even after kidney transplantation [20,21].

Our study has several limitations, comparable to other studies in this field. Since the study was conducted at a single clinical centre and the exclusion criteria were strict, this resulted in a relatively small number of participants. Furthermore, it consisted only of Caucasian individuals, making extrapolation to other countries or populations partially limited. Additionally, we measured serum endocan levels only once, whereas serial monitoring of serum endocan levels may provide more prognostic information. In future studies, potential exclusion of medications that may interfere with endothelial integrity could also prove insightful. Despite these limitations, the unique focus on a non-diabetic population strengthens the relevance of these findings. We believe our study meaningfully contributes to the ever-expanding body of evidence linking serum endocan levels to CKD progression and adverse cardiovascular outcomes.

## 5. Conclusions

Our study explored and confirmed the association between elevated serum endocan levels in non-diabetic HD patients and the occurrence of MACEs and mortality and positions endocan as a cardiovascular risk factor, which can also serve as important biomarker reflecting endothelial dysfunction as a critical contributor to cardiovascular morbidity in ESKD. Conducting larger-scale, multicentre clinical trials that emphasise frequent follow-up could enhance our understanding of the applicability of endocan as a prognostic and diagnostic marker in kidney diseases, potentially even defining relevant cutoff values for its serum levels for timely identification of patients with the highest cardiovascular risk.

## Figures and Tables

**Table 1:** Demographic, clinical, and biochemical variables.

Parameters	1st Tercile of Serum Endocan Levels (2.99–4.67 µg/L)	2nd Tercile of Serum Endocan Levels (4.68–6.29 µg/L)	3rd Tercile of Serum Endocan Levels (6.30–7.93 µg/L)	*p*-Value
Number of patients	25 (33.3%)	25 (33.3%)	25 (33.3%)	
Sex				0.214
Male	18 (72.0)	14 (56.0)	12 (48.0)	
Female	7 (28.0)	11 (44.0)	13 (52.0)	
Age (years)	50.00 [41.00, 64.00]	71.00 [64.00, 75.00]	77.00 [72.00, 81.00]	<0.001
HD vintage (months)	31.00 [6.00, 96.00]	53.00 [21.00, 109.00]	62.00 [48.00, 97.00]	0.083
Interdialytic weight gain (kg)	2.56 (1.06)	2.28 (0.99)	2.44 (1.03)	0.629
Body mass index (kg/m^2^)	27.39 [24.40, 30.44]	25.91 [22.50, 28.22]	26.40 [23.20, 29,70]	0.448
Diabetes mellitus	0 (0.0)	0 (0.0)	0 (0.0)	1.000
Vascular access				0.027
Central venous catheter	6 (24.0)	15 (60.0)	13 (52.0)	
AV fistula	19 (76.0)	10 (40.0)	12 (48.0)	
Follow up (months)	60.00 [60.00, 60.00]	38.00 [20.00, 60.00]	23.00 [14.00, 32.00]	<0.001
Dialysis dose (Kt/V)	1.61 (0.19)	1.66 (0.18)	1.67 (0.20)	0.491
Haematological counts				
Leucocytes (WBCs) (×10^9^/L)	7.00 (1.86)	6.75 (2.08)	7.34 (2.25)	0.596
Erythrocytes (RBCs) (×10^12^/L)	3.78 (0.46)	3.68 (0.43)	3.60 (0.43)	0.342
Haemoglobin concentration (g/L)	109.64 (10.24)	105.88 (9.50)	106.64 (8.26)	0.330
HbA1c (%)	4.8 [4.6, 5.1]	4.8 [4.5, 4.9]	4.9 [4.4, 5.0]	0.598
Thrombocytes (×10^9^/L)	214.00 [178.00, 238.00]	189.00 [177.00, 275.00]	174.00 [135.00, 226.00]	0.369
Serum biochemical parameters				
CRP (mg/dL)	5.50 [4.50, 8.00]	7.80 [4.00, 13.42]	17.60 [11.80, 24.40]	<0.001
Albumin (g/L)	39.00 [36.70, 40.30]	36.60 [34.30, 37.90]	37.10 [35.20, 39.00]	0.026
Glucose (mmol/L)	4.80 [4.40, 5.10]	4.50 [4.30, 4.90]	4.70 [4.40, 5.00]	0.475
Iron (mmol/L)	10.20 [9.30, 12.10]	9.30 [7.60, 14.20]	8.90 [6.50, 11.10]	0.091
Ferritin (ng/mL)	289.00 [123.40, 437.50]	437.60 [252.70, 687.60]	434.90 [286.80, 546.00]	0.070
Total cholesterol (mmol/L)	4.53 (1.13)	4.43 (1.13)	3.96 (1.26)	0.196
LDL cholesterol (mmol/L)	2.12 [1.89, 2.67]	2.12 [1.76, 2.35]	1.87 [1.43, 2.23]	0.236
Calcium (mmol/L)	2.23 [2.07, 2.34]	2.33 [2.18, 2,41]	2.21 [2.04, 2.30]	0.139
Phosphorus (mmol/L)	1.77 (0.37)	1.63 (0.45)	1.58 (0.39)	0.491
PTH (pg/mL)	222.00 [112.00, 307.00]	348.00 [253.00, 423.00]	283.00 [162.00, 403.00]	0.085
Endocan (µg/L)	4.34 (0.47)	5.73 (0.36)	6.79 (0.40)	<0.001
Therapy:				
RAAS inhibitor (%)	15 (60.0)	19 (76.0)	15 (60.0)	0.390
Calcium channel blocker (%)	16 (64.0)	11 (44.0)	15 (60.0)	0.321
Beta blocker (%)	16 (64.0)	15 (60.0)	12 (48.0)	0.492
Statin (%)	15 (60.0)	17 (68.0)	15 (60.0)	0.796
Cause of ESKD:				0.447
Glomerulonephritis (%)	7 (28.0)	6 (24.0)	8 (32.0)	
Arterial hypertension (%)	5 (20.0)	7 (28.0)	9 (36.0)	
Chronic pyelonephritis (%)	3 (12.0)	4 (16.0)	3 (12.0)	
Polycystic kidney disease (%)	4 (16.0)	2 (8.0)	0 (0.0)	
Tubulointerstitial nephritis (%)	0 (0.0)	3 (12.0)	2 (8.0)	

HD: haemodialysis, AV: arteriovenous fistula, WBCs: white blood cells; RBCs: red blood cells; CRP: C-reactive protein, PTH: parathyroid hormone, RAAS: renin angiotensin aldosterone system, and ESKD: end-stage renal disease.

**Table 2:** Parameters of peripheral and central haemodynamics.

Parameters	1st Tercile of Serum Endocan Levels (2.99–4.67 µg/L)	2nd Tercile of Serum Endocan Levels (4.68–6.29 µg/L)	3rd Tercile of Serum Endocan Levels (6.30–7.93 µg/L)	*p*-Value
PWV (m/s)	8.17 (1.22)	10.46 (1.64)	12.62 (1.51)	<0.001
PWV after HD (m/s)	7.88 (1.57)	10.36 (1.64)	11.94 (1.45)	<0.001
Peripheral SBP (mmHg)	143.00 [137.00, 152.00]	152.00 [125.00, 164.00]	157.00 [149.00, 173.00]	0.026
Peripheral DBP (mmHg)	88.20 (14.07)	87.36 (14.98)	93.28 (14.83)	0.308
MAP (mmHg)	112.00 [98.00, 122.00]	117.00 [100.00, 121.00]	120.00 [114.00, 134.00]	0.049
PP (mmHg)	55.40 (8.09)	62.64 (19.62)	69.88 (19.59)	0.012
HR (per minute)	75.00 [72.00, 79.00]	71.13 [64.00, 74.00]	70.00 [60.00, 78.00]	0.151
AI	23.48 (8.23)	27.36 (15.05)	38.56 (10.38)	<0.001
Central SBP (mmHg)	130.00 [126.00, 141.00]	133.00 [118.00, 148.00]	146.00 [131.00, 163.00]	0.024
Central DBP (mmHg)	88.12 (15.57)	87.64 (15.58)	94.60 (15.24)	0.213
Central PP (mmHg)	43.00 [39.00, 47.00]	46.00 [38.00, 49.00]	51.00 [44.00, 69.00]	0.029
Peripheral SBP after HD (mmHg)	130.00 [120.00, 140.00]	142.00 [125.00, 162.00]	149.00 [131.00, 158.00]	0.059
Peripheral DBP after HD (mmHg)	84.16 (14.82)	84.60 (19.17)	86.24 (14.97)	0.895
MAP after HD (mmHg)	100.00 [92.00, 116.00]	104.00 [96.00, 127.00]	117.00 [104.00, 125.00]	0.143
PP after HD (mmHg)	52.00 [41.00, 58.00]	58.00 [47.00, 67.00]	61.00 [48.00, 73.00]	0.124
HR after HD (per minute)	83.52 (14.41)	77.91 (11.52)	75.47 (12.79)	0.087
AI after HD	18.00 [13.00, 32.00]	30.00 [15.00, 40.00]	30.00 [19.00, 43.00]	0.036
Central SBP after HD (mmHg)	124.64 (17.96)	131.88 (22.78)	133.60 (19.55)	0.257
Central DBP after HD (mmHg)	84.64 (16.58)	85.20 (20.22)	88.20 (14.80)	0.738
Central PP after HD (mmHg)	42.00 [30.00, 50.00]	47.00 [34.00, 55.00]	44.00 [33.00, 53.00]	0.461

PWV: pulse wave velocity, SBP: systolic blood pressure, DBP: diastolic blood pressure, MAP: mean arterial pressure, PP: pulse pressure, HR: heart rate, and AI: augmentation index.

**Table 3:** Association between serum endocan levels and observed outcomes.

Parameters	1st Tercile of Serum Endocan Levels (2.99–4.67 µg/L)	2nd Tercile of Serum Endocan Levels (4.68–6.29 µg/L)	3rd Tercile of Serum Endocan Levels (6.30–7.93 µg/L)	*p*-Value
Number of patients	25 (33.3)	25 (33.3)	25 (33.3)	
Number of patients who experienced a major adverse cardiovascular event (MACE) (%)	3 (12.0)	9 (36.0)	20 (80.0)	<0.001
Cause of death:				<0.001
not applicable (surviving participants) (%)	21 (84.0)	8 (32.0)	0 (0.0)	
MACE (%)	1 (4.0)	8 (32.0)	20 (80.0)	
other causes (%)	3 (12.0)	9 (36.0)	5 (20.0)	

**Table 4:** Detailed results of the Cox multivariate model.

Outcome	MACE		MACE-Related Mortality		All-Cause Mortality	
Variable	HR (95%-CI)	*p*-Value	HR (95%-CI)	*p*-Value	HR (95%-CI)	*p*-Value
Serum endocan concentration	4.09 (1.72–9.74)	0.00144	2.64 (1.23–5.65)	0.0125	1.86 (1.07–3.23)	0.0272
Age	1.01 (0.96–1.07)	0.711	1.02 (0.97–1.08)	0.386	1.03 (0.99–1.08)	0.11
Sex (male)	1.96 (0.87–4.41)	0.103	1.04 (0.46–2.37)	0.921	1.30 (0.67–2.52)	0.434
Duration of haemodialysis	1.01 (1.00–1.01)	0.0688	1.00 (1.00–1.01)	0.284	1.01 (1.00–1.01)	0.00778
Serum albumin concentration	0.98 (0.85–1.12)	0.736	1.05 (0.91–1.22)	0.504	0.99 (0.89–1.10)	0.824
BMI	1.00 (0.93–1.07)	0.997	0.99 (0.92–1.06)	0.751	0.99 (0.93–1.05)	0.638
Central systolic pressure	0.99 (0.98–1.01)	0.453	1.00 (0.99–1.02)	0.731	1.00 (0.99–1.02)	0.48
Augmentation index	1.01 (0.98–1.04)	0.7	1.02 (0.99–1.05)	0.253	1.00 (0.98–1.03)	0.926

MACE: major adverse cardiovascular event, HR: hazard ratio, and BMI: body mass index.

## Data Availability

The dataset used, analysed, or generated in this research is available from the corresponding author (E.O.) upon reasonable request.

## Abbreviations

The following abbreviations are used in this manuscript:

CKD	chronic kidney disease
CVD	cardiovascular disease
HD	haemodialysis
DKD	diabetes mellitus
MACE	major adverse cardiovascular event
AVF	arteriovenous fistulas
TCVC	tunnelled central venous catheter
ESKD	end-stage kidney disease
FMD	flow-mediated dilation
IgA	immunoglobulin A
PD	peritoneal dialysis
sICAM-1	intercellular adhesion molecule-1
sVCAM-1	vascular adhesion molecule-1
hs-CRP	high-sensitivity CRP
CIMT	carotid intima–media thickness
PWV	pulse wave velocity
AI	augmentation index
MAP	mean arterial pressure
PP	pulse pressure
SBP	systolic blood pressure
DBP	diastolic blood pressure
HR	heart rate
PTH	parathyroid hormone
BCM	bioimpedance method
ELISA	enzyme-linked immunosorbent assay
RAAS	renin angiotensin aldosterone system
